# Clinical Outcomes and Correlation With Biochemical Control in Hydroxocobalamin‐Treated Patients With Early‐Onset Cobalamin C Disease

**DOI:** 10.1002/jmd2.70091

**Published:** 2026-04-27

**Authors:** Arthavan Selvanathan, Ashley Hertzog, Jacqui Russell, Rosie Junek, Carolyn Ellaway, Kate Lichkus, Adviye Ayper Tolun, Kaustuv Bhattacharya

**Affiliations:** ^1^ Genetic Metabolic Disorders Service Sydney Children's Hospitals Network Sydney New South Wales Australia; ^2^ Faculty of Medicine and Health University of Sydney Sydney New South Wales Australia; ^3^ NSW Biochemical Genetics Service the Children's Hospital at Westmead, Sydney Children's Hospitals Network Sydney New South Wales Australia; ^4^ NSW Newborn Screening Programme the Children's Hospital at Westmead, Sydney Children's Hospitals Network Sydney New South Wales Australia; ^5^ Faculty of Medicine and Health University of New South Wales Sydney New South Wales Australia

**Keywords:** biomarkers, cobalamin C disease, hydroxocobalamin, inborn errors of metabolism, maculopathy, neurocognitive outcomes

## Abstract

Cobalamin C (cblC) disease is the most common disorder of Vitamin B12 activation. The early‐onset form presents within the first few months of life, with some patients identified through newborn screening (NBS). However, despite early detection and optimal treatment, patient outcomes remain poor, with intellectual impairment and progressive visual loss in the majority. We reviewed a cohort of 10 patients with cblC disease, all identified either by NBS or a neonatal clinical presentation. We reviewed their biochemical control and correlated this with clinical progress and treatment. The majority of the cohort (including four asymptomatic patients) was identified through NBS and had genotypes predictive of early‐onset disease. Clinical outcomes improved with standard‐of‐care treatment (hydroxocobalamin, betaine, and folinic acid) but were suboptimal, with both neurocognitive (6/10) and ophthalmological manifestations (10/10) occurring in most patients. One patient died at 5 months of age, and it is unclear whether this was related to cblC disease or not. Across over 250 timepoints from 9 patients, there was minimal correlation between cumulative intramuscular hydroxocobalamin (OHCbl) dose and biomarkers, including methylmalonic acid (*r* ^2^ = 0.0031) and total homocysteine (*r* ^2^ = 0.2858) levels. This study provides comprehensive, longitudinal biochemical, and clinical follow‐up of patients with cblC disease treated from soon after birth, often presymptomatically. Our findings corroborate previous observations regarding the lack of correlation of current biomarkers, both with disease progression and with standard (< 0.3 mg/kg/day) hydroxocobalamin dosing. Further investigation of the clinical impact of early high‐dose OHCbl treatment is needed in larger cohorts of patients.

## Key Points

- Current biomarkers for cobalamin C disease are diagnostically useful, but correlate poorly with both disease progression and hydroxocobalamin dosing in the standard range (< 0.3 mg/kg/day).

## 1 Introduction

Cobalamin C (cblC) disease is the most common inborn error of Vitamin B12 metabolism. It is caused by biallelic pathogenic variants in the *MMACHC* gene, which results in an inability to convert Vitamin B12 into both of its biologically active forms, adenosylcobalamin, and methylcobalamin [1]. Adenosylcobalamin is the cofactor for the enzyme methylmalonyl‐CoA mutase, and methylcobalamin is the cofactor for methionine synthase. As such, the biochemical hallmarks of the disease are methylmalonic acidaemia, hyperhomocysteinaemia, and low serum methionine [2].

The clinical phenotypes of cblC disease can be divided based on the age of onset; however, there is substantial overlap between the groups. These phenotypes include an in utero presentation (nonimmune hydrops, intrauterine growth restriction); a neonatal presentation (encephalopathy, poor feeding, progressive microcephaly); an infantile/early childhood presentation (poor growth, developmental delay, seizures, hypotonia, cytopenias, maculopathy); and an adolescent/adult presentation (neuropsychiatric symptoms, thromboembolic phenomena, progressive cognitive impairment) [3].

The outcomes for treated patients who presented symptomatically with the earlier‐onset forms of cblC disease have been well‐studied. Large case series indicated high mortality in those with onset in the first year of life, with neurocognitive impairment and maculopathy in almost all survivors despite treatment with hydroxocobalamin (OHCbl) [4, 5]. However, it is more difficult to determine outcomes for patients treated presymptomatically when identified by newborn screening (NBS) programs. Bourque and colleagues compared 11 patients diagnosed clinically with neonatal‐onset cblC disease with 15 patients diagnosed while asymptomatic (identified through NBS or due to a positive family history). Sixty percent of patients who were diagnosed and treated in the presymptomatic period had developmental delay and 33% had eye disease. It is worth noting that only 2 of the 15 patients in the NBS group from this study had genotypes known to be associated with severe, early‐onset disease [6].

From a molecular perspective, there is some degree of genotype–phenotype correlation that has been established. Morel and colleagues reviewed 37 published patients with cblC disease and biallelic variants in *MMACHC* [6]. The c.271dupA and c.331C>T variants accounted for 68% of patients who had early‐onset disease (presenting within the first 6 months of life). On the other hand, at least one copy of the c.394C>T variant has been seen in many patients presenting with late‐onset disease [7]. Morel et al. reported one patient who was compound heterozygous for c.271dupA and c.394C>T and presented with early‐onset disease, suggesting that genotype–phenotype correlation is not perfect in this condition.

The evaluation of biochemical parameters such as plasma methylmalonic acid (MMA), total homocysteine (tHcy), and methionine is fundamental for both diagnosis and monitoring the initial response to treatment. Intramuscular OHCbl dosing provides supratherapeutic levels of cobalamin in an attempt to overcome the biochemical defect; betaine provides an alternate pathway for homocysteine re‐methylation (conversion to methionine); folinic acid ensures there is adequate 5‐methyltetrahydrofolate for homocysteine re‐methylation through the endogenous enzyme methionine synthase [1, 2].

While rapid improvement in all three biomarkers is typically observed when these therapies are initiated at diagnosis, the plasma MMA and tHcy never normalize in early‐onset forms of cblC disease. Higher concentrations of plasma MMA and tHcy are seen in other disorders (such as homocystinuria and isolated methylmalonic acidaemia), but these conditions do not have the same range of neurocognitive and ocular manifestations that are seen with *MMACHC* deficiency [8]. It is important to confirm whether there is a correlation between these established biomarkers and treatment dosing, best assessed through longitudinal follow‐up of a cohort of patients diagnosed in infancy.

## 2 Aims

We retrospectively reviewed clinical, biochemical, and molecular data from all 10 patients diagnosed with cblC disease in New South Wales, Australia since the commencement of expanded NBS in 1998. We assessed the impact of treatment on established plasma biomarkers (MMA, tHcy, and methionine), as well as whether these biomarkers predicted clinical severity.

## 3 Methods

### 3.1 Inclusion and Exclusion Criteria

Patients were identified through the Genetic Metabolic Disorders Service (GMDS), Sydney Children's Hospitals Network (SCHN) database, and cross‐checked with the NSW Newborn Screening Program database. Patients were considered eligible for inclusion in the study if they had a confirmed diagnosis of cblC disease, either by molecular testing or complementation studies; were less than 18 years of age at the time of cohort identification; and received both treatment and biochemical monitoring. Patients diagnosed before the commencement of expanded NBS in 1998 were excluded.

### 3.2 Ethics Approval and Consent

Ethics approval was received from the SCHN Human Research Ethics Committee (HREC) for retrospective chart review of patient notes and blood results (Approval Number CCR2023/14), and the legal guardians of all patients consented for this chart review to proceed. The medical records were accessed to collect epidemiological and diagnostic information, longitudinal clinical, and biochemical information as well as details of treatment.

### 3.3 Numerical and Statistical Analysis

For each patient, longitudinal plasma MMA, tHcy, and methionine concentrations (collected in lithium heparin tubes) were tabulated, as well as the corresponding dosing regimen of intramuscular OHCbl, betaine, and folinic acid at the time.

The area under the curve (AUC) functions for all three medications and all three established biomarkers were estimated using the trapezoidal rule for integration [9]. Uneven intervals were used for an improved approximation of the true AUC, with between 15 and 40 intervals for every patient. “Lifetime” AUC values were then standardized per year and compared across patients. The correlation between the AUCs for individual medications and biomarkers was assessed via the *r* ^2^ value.

## 4 Results

### 4.1 Baseline Information

Table 1 outlines baseline information related to the 10 patients from our cohort receiving ongoing follow‐up. Patient 10's clinical history is reported separately below given they were deceased at 5 months of age.

**TABLE 1:** Baseline clinical information for cohort of patients with cblC disease.

Patient	1	2	3	4	5	6	7	8	9	10
Gender	Female	Male	Female	Female	Female	Male	Female	Male	Male	Male
Age at diagnosis	10 days	22 days	1 month	14 days	16 days	15 days	12 days	3 months	9 days	14 days
Family history (Y/N)	N	Y	N	N	N	N	N	N	N	N
Consanguinity	Y	Y	Y	N	N	Y	N	Y	N	N
Birth weight centile	2nd	10th	11th	11th	5th	4th	29th	4th	1st	13th
Clinical presentation	Lethargy 1 day post NBS diagnosis	Lethargy with poor feeding 2 days prior to NBS recall; seizures and persistent nystagmus the day after	Poor feeding, followed by hypotonia and seizures, with communicating hydrocephalus	Born at 34 weeks, NBS diagnosis	NBS diagnosis	NBS diagnosis; slightly lethargic on first examination	NBS diagnosis	Presented at 2 months of age with abnormal movements of his eyes and upper limbs	NBS diagnosis	NBS diagnosis
Molecular analysis of the *MMACHC* gene	c.271dup homozygous	c.271dup homozygous	c.271dup homozygous	c.271dup homozygous	c.82‐1G>A and c.271dup	c.271dup homozygous	c.217C>T and c.567dup	c.394C>T homozygous	c.271dup and c.331C>T	c.271dup and c.388&lowbar;390del
C3 NBS (< 8)	6.2	9.7	4.7	8.8	4.8	7	10.7	10.6	13.7	7.5
C3/C2 NBS (< 0.23)	0.13	0.18	0.09	0.38	0.24	0.43	0.33	0.22	0.29	0.25
Plasma MMA (μmol/L: RR < 1.57) at diagnosis	197.7	50	61.78	3.05	129.45	129	151	18	81.89	182
Plasma total homocysteine (μmol/L: RR 3.3–19.2) at diagnosis	174.4	259.7	250.7	38.9	164.3	232.7	234.2	121	196.6	182
Plasma methionine (μmol/L: RR 12–43) at diagnosis	6	2	4	20	9	3	4	5	4	8

All patients were diagnosed within the first few months of life, with 7 of 10 patients identified by NBS. Of 10 patients, 5 patients were asymptomatic at the time of diagnosis. Interestingly, 2 of the 3 patients with the most severe clinical presentations (Patients 3 and 8) were not identified through NBS, though this may reflect subacute accumulation of metabolites over time. All patients were treated with a combination of intramuscular OHCbl, oral betaine, and oral folinic acid. No patients were managed with dietary protein restriction.

Four patients had a homozygous c.271dup genotype, with another patient compound heterozygous for c.271dup in combination with c.331C>T, all predictive of severe and early‐onset disease [6, 7]. One patient is homozygous for the c.394C>T variant that has been associated with late‐onset disease [6]; however, their clinical presentation at 2 months of age (with abnormal eye and upper limb movements of age) is in keeping with early‐onset disease. Patient 5 carries the c.271dup in a compound heterozygous state with c.82‐1G>A. The latter variant has been reported in only a limited number of cases [7, 10], and it is therefore difficult to predict its associated phenotype. Patient 7 was diagnosed prior to the availability of DNA sequencing for *MMACHC* and her diagnosis was made via complementation studies. Retrospectively, she was noted to have two previously‐reported variants: an early truncating variant (c.217C>T causing p.Arg73*) and a frameshift variant creating a transcript that may escape nonsense‐mediated decay (c.567dup causing p.Ile190Tyrfs*13).

Patient follow‐up ranged from 5 months to 15 years with a median length of follow‐up 6 years, and all were treated from the time of diagnosis.

### 4.2 Biochemical Outcomes

Diagnostic and longitudinal biochemical data are presented in Table S1. All patients had elevated plasma MMA and tHcy at the time of diagnosis (median 105.7 and 189.1 μmol/L, respectively), and 9 of 10 patients had low plasma methionine levels. While the average plasma methionine level over time was within the normal range for all patients, there was variability in the average MMA level (1.6–31.9 μmol/L), though these levels are substantially lower compared to those seen in classical methylmalonic acidaemia [11]. There was similar variability in tHcy (28.8–102.8 μmol/L), though most patients had levels below 50 μmol/L.

### 4.3 Clinical Outcomes

Despite the biomarker concentrations while on therapy suggesting adequate management (or at least what would be considered excellent control for the individual conditions, isolated methylmalonic aciduria and classical homocystinuria), the clinical effects of disease were marked (Table 2). Of 10 patients, 7 patients had some degree of developmental delay; one had borderline cognitive impairment, one was too young to be formally assessed, and the remaining patient was assessed as having normal cognition. All patients had at least one ophthalmological manifestation (six with bull's eye maculopathy, five with nystagmus, three with an esotropia). None of the patients had normal visual acuity. Cytopenias were present in three patients and co‐existing iron‐deficiency anemia was also common (30%). Of 10 patients, 9 patients had normal growth parameters.

**TABLE 2:** Clinical outcomes in cohort of patients with cblC disease.

Patient	1	2	3	4	5	6	7	8	9	10
Age at last follow‐up	13 years	9 years	4 years	8 years	6 years	13 years	17 years	14 years	6 years	5 months (deceased)
Age first walked	16 months	16 months	19 months	16 months	14 months	17 months	15 months	14 months	24 months	N/A
Age first words	16 months	12 months	16 months	25 months	11 months	16 months	10 months	20 months	18 months	N/A
Most recent developmental assessment	Not performed.	Mild‐moderate intellectual impairment, with particular weakness in receptive and expressive language.	Moderate global developmental delay; strengths in socialization and persistence with tasks; weakness in receptive and expressive language.	Mild intellectual impairment, with particular weakness in expressive language. Diagnosed with ADHD.	Borderline cognition: low‐average to average developmental domains and adaptive function. Diagnosed with autism and anxiety.	Global developmental delay, autism spectrum disorder.	Not formally assessed: mild learning difficulties. Difficulties with balance.	Mild intellectual disability, adaptive function extremely low.	Mild global developmental delay.	Not performed.
Educational attainment	Year 7 in a mainstream class: utilizes braille with additional learning support.	In Year 3 in a learning support unit.	N/A	Year 2, in a mainstream class with teacher's aide support.	In preschool: has some additional support for emotional regulation and visual difficulties.	Year 7 in a learning support unit.	Year 11 in a mainstream class.	Year 8 in a learning support unit.	Year 1 in a mainstream class with some learning difficulties.	N/A
Eye Abnormalities	Bilateral severe bulls eye maculopathy.	Bilateral severe bulls eye maculopathy, horizontal nystagmus.	Intermittent esotropia, nystagmus, early stages of bulls eye maculopathy.	Left divergent squint with slight hypermetropia (managed with patching).	Stable bull's eye maculopathy, nystagmus when fatigued, poor depth perception.	Right amblyopia secondary to right exotropia, bilateral bulls eye maculopathy with pigmentary retinopathy.	Bilateral maculopathy causing decreased visual acuity.	Horizontal nystagmus (bidirectional).	Mild horizontal nystagmus (bidirectional).	Pigmentary retinopathy with nystagmus: able to fix but following only intermittently.
Visual acuity (*R* = right, L = left)	R:6/120, L:10/120	R:6/120, L:6/120	R:6/38, L:6/38	R:6/12, L:6/15	R:6/76, L:6/76	R:6/38, L:6/30	R:6/38, L:6/30	R:6/30, L:6/30	R:6/19, L:6/19	Not available
Thromboembolic disease	N	N	Hydrocephalus in the neonatal period	N	N	N	N	N	N	N
Hematological abnormalities	N	Initial mild anemia (Hb 90g/L, resolved by 4 months of age).	Pancytopenia at the time of diagnosis.	N	N	Intermittent iron‐deficiency anemia.	N	Intermittent mild iron‐deficiency anemia; cytopenias at 5 months of age (self‐resolved).	Iron‐deficiency anemia.	N
Most recent height (centile)	60	76	83	12	68	1	46	24	47	Not available
Most recent weight (centile)	89	95	70	14	89	1	92	23	97	25
Most recent head circumference (centile)	67	88	24	7	75	25	59	27	30	Not available
Other co‐morbid conditions	Vitamin D deficiency.	—	Hydrocephalus with subsequent shunt blockage requiring revision.	—	Virus‐induced wheeze.	Vitamin D deficiency.	Protein‐losing enteropathy at 12 months of age (resolved); episode of post‐viral acute cerebellitis at 15 years of age, one episode of unexplained encephalopathy.	—	Single complex febrile seizure	Two episodes of pneumonia (at age 2 weeks and 18 weeks). Poor feeding with suspected swallowing dysfunction.

Patient 10 was the only patient who died from this cohort. He had no neonatal symptoms of cblC disease, though he did develop abnormal eye movements at 3.5 months of age despite intramuscular OHCbl treatment (1 mg every second day), betaine and folinic acid. He had hypotonia, pigmentary retinopathy and possible oropharyngeal dysfunction. At 5 months of age, he was found unresponsive in his cot, 30 min after he appeared to be sleeping and breathing peacefully. Postmortem analysis identified mildly delayed cerebral myelination, but no other abnormalities. The patient's death was thought to be consistent with sudden infant death syndrome, though it is possible that it was an unexpected complication of cblC disease.

### 4.4 Effect of Treatment on Outcomes

All patients in this cohort received treatment with intramuscular OHCbl, betaine, and folinic acid. This was commenced either at the time of NBS identification or within 2 weeks of symptom onset if diagnosed clinically. The dosing of OHCbl, with two exceptions (Patients 3 and 4), ranged from 0.15 to 1.3 mg/kg/week (equivalent to 0.02–0.2 mg/kg/day). Patient 10 was not included in the analysis because of limited datapoints (only three sets of blood tests, with three follow‐up appointments prior to their death at 5 months of age). A full list and graphs of individual datapoints for each patient, including biochemical indices and contemporaneous medication dosing, are available in Document S2.

**FIGURE 1:**
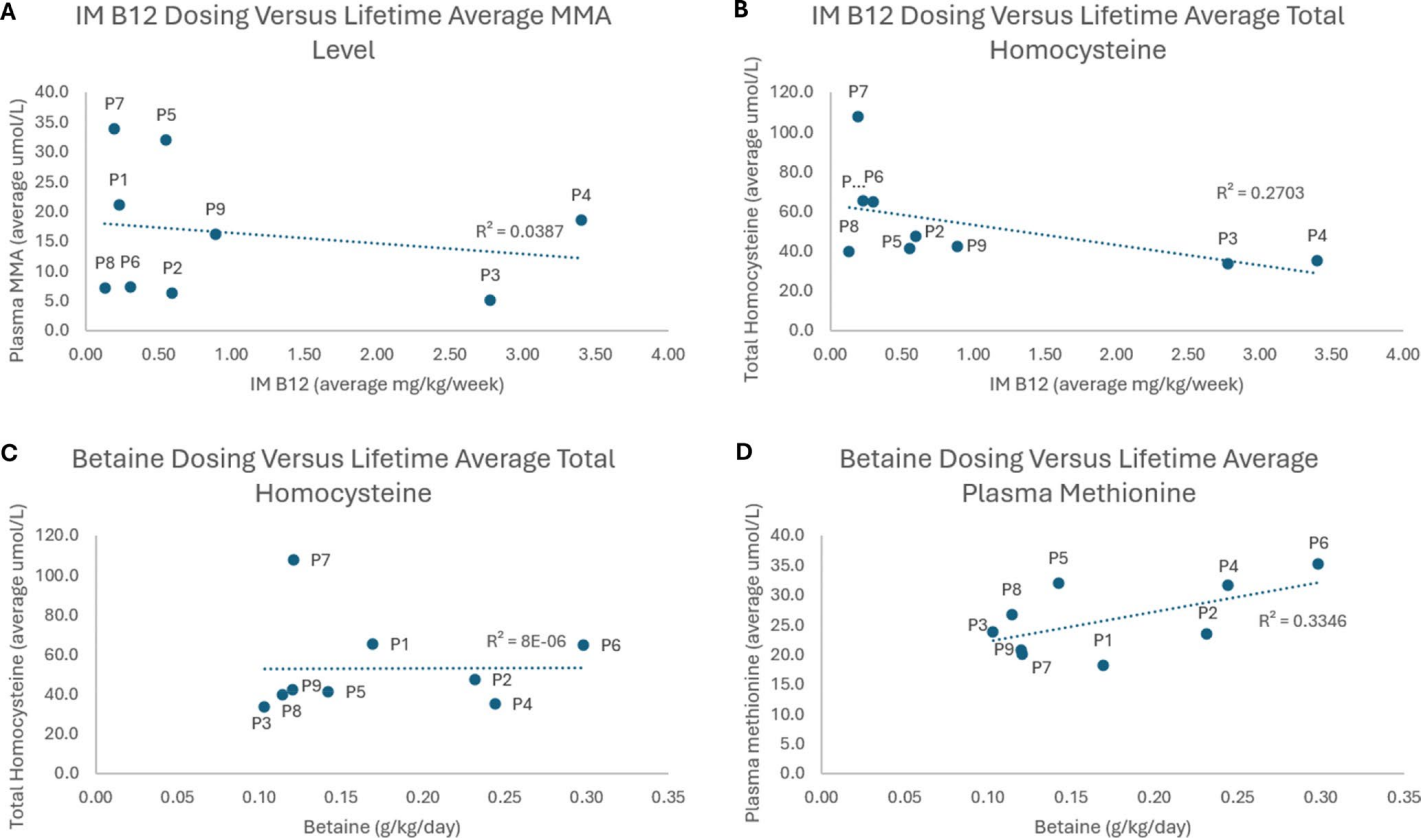
Lack of correlation between standard treatments and established biomarkers in patients with cobalamin C disease. (A) Intramuscular B12 versus MMA; (B): intramuscular B12 versus total homocysteine; (C) betaine versus total homocysteine; (D) betaine versus plasma methionine.

Figure 1 demonstrates the poor correlation between intramuscular OHCbl dosing and average plasma MMA levels (*r* ^2^ = 0.032). This suggests that OHCbl dosing of < 0.3 mg/kg/day is only partially able to suppress MMA production. There was a slight but poor negative correlation between intramuscular OHCbl dosing and tHcy levels (*r* ^2^ = 0.2663). Similarly, the dosing of betaine did not correlate with tHcy levels: this was most obvious in Patient 6 where tHcy levels remained in the 50–80 μmol/L range despite dose escalation up to 0.5 g/kg/day (Document S2). Plasma methionine levels generally normalized from the time of commencement of treatment.

## 5 Discussion

CblC disease was first described in 1969 by Mudd and colleagues, in a child noted to have both high plasma MMA and tHcy at 4 weeks of age, with low serum (and cerebral) methionine [12]. This infant died at 7 weeks of age, and it was postulated at the time that intramuscular OHCbl and betaine could form part of the management of this novel condition.

These standard biomarkers for cblC disease are well‐established, and there is clear evidence for treatment with intramuscular OHCbl and betaine [1, 2, 3]. However, our data suggest that in the early‐onset form of the condition, long‐term biochemical control does not correlate with disease progression and that treatment generally improves but does not normalize biochemical parameters. This lack of correlation is apparent even in the 6 patients that had minimal symptoms at the time of their diagnosis through NBS. Patient 7 illustrates this particularly well: she was identified through NBS and had marked elevations in plasma MMA and tHcy at diagnosis, with decreased serum methionine. Her long‐term control was also poor, with an average lifetime plasma MMA of 31.9, tHcy of 102.8 μmol/L and periods of hypomethioninaemia. Despite this, her long‐term outcome has been generally positive in comparison to other patients, having been in mainstream schooling throughout childhood (though she recently had an episode of acute encephalopathy of unclear etiology) and developing only mild maculopathy.

### 5.1 Need for Novel Biomarkers

The molecular defect in cblC disease leads to impaired cobalamin activation, with consequent accumulation of plasma MMA and tHcy, and reduced methionine. These biomarkers are relevant for disease diagnosis, and their reduction prevents acute metabolic decompensation; however, patients still often have neurocognitive and ophthalmological challenges despite good biochemical control. It is important that novel biomarkers are developed that correlate with outcomes in these domains.

There are studies looking at novel biomarkers for disorders affecting the two basic biochemical pathways in cblC disease (methylmalonic acidaemia and hyperhomocysteinaemia). In mutase‐deficient methylmalonic aciduria, there is evidence of secondary mitochondrial dysfunction leading to increased production of reactive oxygen species. Chandler and colleagues assessed mitochondrial ultrastructure and function in a Mut−/− mouse model and noted megamitochondria on electron microscopy of hepatic and renal tubular cells, with evidence of respiratory chain dysfunction and reduced intracellular glutathione [13]. In this regard, mitochondrial biomarkers such as FGF21 and GDF15 could become relevant markers for elements of the disease unexplained by MMA/tHcy/methionine levels (maculopathy in particular), as has been suggested for isolated methylmalonic acidaemia [14].

There is also evidence that increased tHcy levels lead to increased reactive oxygen species generation. This is thought to lead to osteoclast activation, causing the osteoporosis that is commonly seen in patients with even mild increases in tHcy [15].

As cblC disease results in accumulation of both MMA and tHcy, one might expect similar disruptions to the citric acid cycle and increased reactive oxygen species in the mitochondria. Hence, it is unsurprising that increased reactive oxygen species generation has been reported in cblC disease [16, 17].

More recent work by Head and colleagues (2023) suggested that some of the secondary mitochondrial dysfunction may be secondary to hyperacylation (particularly excessive methylmalonylation) of key proteins involved in the citric acid cycle, the urea cycle, mitochondrial DNA replication and glutathione metabolism among others [18]. They proposed that sirtuin, specifically *SIRT5*, activity was often concomitantly low in mouse models of mutase‐deficient methylmalonic aciduria, and that therapeutic provision of a hyperacylation‐resistant SIRT5 protein resulted in a therapeutic effect independent of MMUT activity.

Forny and colleagues approached the same question regarding the pathophysiology of mutase‐deficient methylmalonic aciduria through an unbiased multi‐omics platform, and confirmed secondary reduction in citric acid cycle intermediates, in turn causing insufficiency of anaplerosis [19]. Unfortunately, this has not been replicated in patient cohorts with cblC disease, though we did not see an increase in one of the mentioned Krebs cycle intermediates (alpha‐ketoglutarate) in the diagnostic urine samples of any of our patients.

### 5.2 Need for Novel Treatment Strategies

Historically, patients at our center diagnosed in the pretandem mass spectrometry NBS era (before 1998) were treated with second‐daily, weekly, or even three‐weekly dosing of OHCbl. After the death of Patient 10, the dosing frequency in all patients was increased to at least once per week (with three having daily injections). Of the patients on daily injections, Patient 2 was transitioned onto this regimen at 7.5 years of age (after significant progression of maculopathy), whereas the other two (Patients 3 and 4) have been on daily dosing throughout life.

Despite this change in dosing, similar to other cohorts worldwide [20], our data demonstrate that standard‐dose OHCbl is insufficient to prevent neurocognitive delay and eye disease in almost all patients. Additionally, the average dosing of OHCbl does not correlate with the average plasma MMA or tHcy levels over time. While this may be due to variable timing of sample collection, it also raises questions about the reliability of these biomarkers as indicators of disease progression. In these patients, the magnitude of response in plasma MMA is similar to patients with Cobalamin A deficiency, but the clinical complications are quite different, with most of the latter patients having an enduring clinical and biochemical response [21, 22].

There is evidence in the literature to suggest that high‐dose OHCbl (> 0.3 mg/kg/day) may be required to improve clinical and biochemical outcomes. While the numbers of patients on treatment with high‐dose OHCbl are limited, there have been reports of improved biochemistry [23] and developmental progress [24, 25]. Patients 3 and 4 in our cohort have undergone intramuscular OHCbl dose escalation on parental request (currently taking 0.4 and 1.1 mg/kg/day respectively). Patient 3 has developed mild maculopathy at a young age; however, Patient 4 in particular appears to have preserved vision despite a severe genotype, in keeping with anecdotal case reports internationally.

Prospective longitudinal study of patients following dose escalation of intramuscular OHCbl may be required to confirm whether this therapy is beneficial. If benefit is proven, subsequent newly diagnosed patients could be treated with high‐dose intramuscular OHCbl from a younger age. It is also important, however, to consider the medical and psychological trauma that may occur with daily injections.

### 5.3 Limitations of This Study

Due to the rarity of the condition, our study is limited by the small cohort size. As a result, genotype–phenotype correlations were not explored, and neither were multiple regression models incorporating other prognostic markers. Despite the small patient cohort, we had data (including concentrations of all three biomarkers, mg/kg dosing of OHCbl, betaine and folinic acid) from over 250 timepoints, allowing for robust AUC analyses.

Another limitation is the retrospective nature of the data, preventing adjustment for confounding factors. In particular, undisclosed medication nonadherence could potentially have led to incomplete suppression of MMA and tHcy levels in some patients, and dietary protein intake (not quantified in this study) could also have impacted levels. Dosing frequency of intramuscular OHCbl also varied between participants, ranging from daily injections to weekly. This limitation would be best mitigated through rigorous prospective clinical trials with a defined treatment protocol.

## 6 Conclusion

Despite early detection through NBS and adherence to standard treatment protocols after diagnosis, most patients with cblC disease still develop neurocognitive and ophthalmological complications. Our longitudinal study highlights the persistent disconnect between biochemical control and clinical outcomes, suggesting that current biomarkers are insufficient predictors of disease progression. These findings underscore a need to explore alternative disease mechanisms that may be contributing to the unexplained clinical variability.

## Author Contributions

**Arthavan Selvanathan:** conception and design, ethics submission, collecting data, analysis/interpretation of data, writing manuscript, reviewing manuscript. **Ashley Hertzog:** concept and design, collecting data, writing manuscript, reviewing manuscript. **Jacqui Russell:** collecting data, reviewing manuscript. **Rosie Junek:** collecting data, reviewing manuscript. **Carolyn Ellaway:** collecting data, reviewing manuscript. **Kate Lichkus:** collecting data, reviewing manuscript. **Adviye Ayper Tolun:** analysis/interpretation of data, reviewing manuscript. **Kaustuv Bhattacharya:** conception and design, analysis/interpretation of data, reviewing manuscript.

## Funding

Components of this project were supported by a Small Projects Grant ($20 000) from Kids Research, the research arm of the Sydney Children's Hospitals Network.

## Ethics Statement

Ethics approval was received from the SCHN Human Research Ethics Committee (HREC) for retrospective chart review of patient notes and blood results (Approval Number CCR2023/14). The legal guardians of all patients provided consent for this chart review to proceed. The medical records were accessed to collect epidemiological and diagnostic information, longitudinal clinical and biochemical information as well as details of treatment.

## Conflicts of Interest

The authors declare no conflicts of interest.

## Supporting information


**Data S1:** (Excel spreadsheet): This document provides the raw data for nine patients, focussing on treatment dosing (of intramuscular B12, betaine, and folinic acid) over time as well as biochemical control (plasma methylmalonate, total homocysteine, and plasma methionine). It demonstrates that both at the patient and at the cohort level, there is no correlation between standard biomarkers and treatment dosing in cobalamin C disease.

**Table S1:** Biochemical outcomes and intramuscular hydroxocobalamin (OHCbl) dosing in cohort of patients with cblC disease.

## Data Availability

The data that support the findings of this study are available on request from the corresponding author. The data are not publicly available due to privacy or ethical restrictions.
