# Supplementary material for: Clinical Outcomes and Correlation With Biochemical Control in Hydroxocobalamin‐Treated Patients With Early‐Onset Cobalamin C Disease
Source: JIMD Rep. 2026 Apr 27;67(3):e70091. doi: 10.1002/jmd2.70091 (PMC13120851; doi:10.1002/jmd2.70091)
Supplement: Supplementary file 2 — Table S1: Biochemical outcomes and intramuscular hydroxocobalamin (OHCbl) dosing in cohort of patients with cblC disease. [file JMD2-67-e70091-s002.docx]

**Supplementary Table 1:** Biochemical Outcomes and Intramuscular Hydroxocobalamin (OHCbl) Dosing in Cohort of Patients with cblC Disease

**
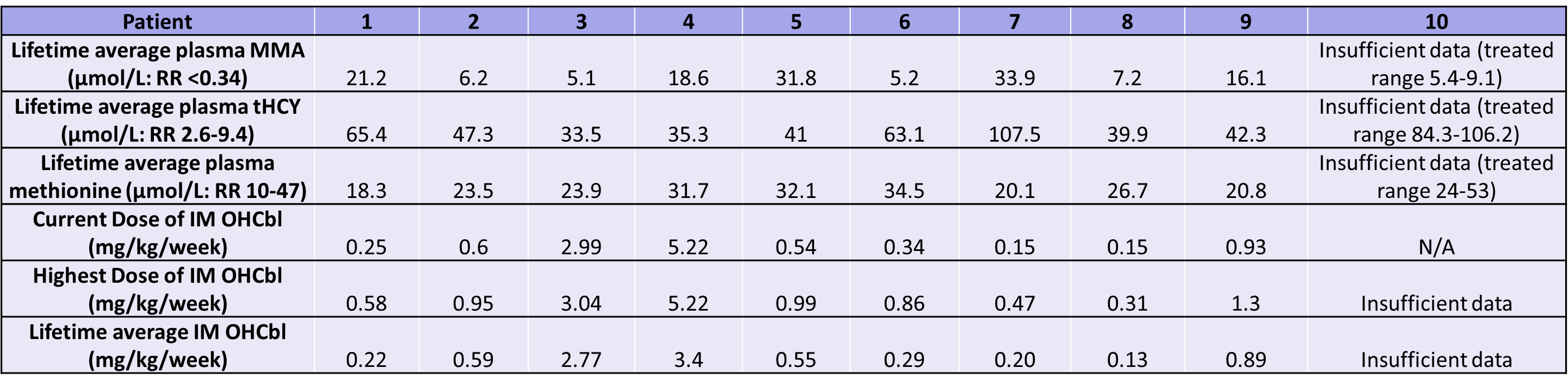
**
